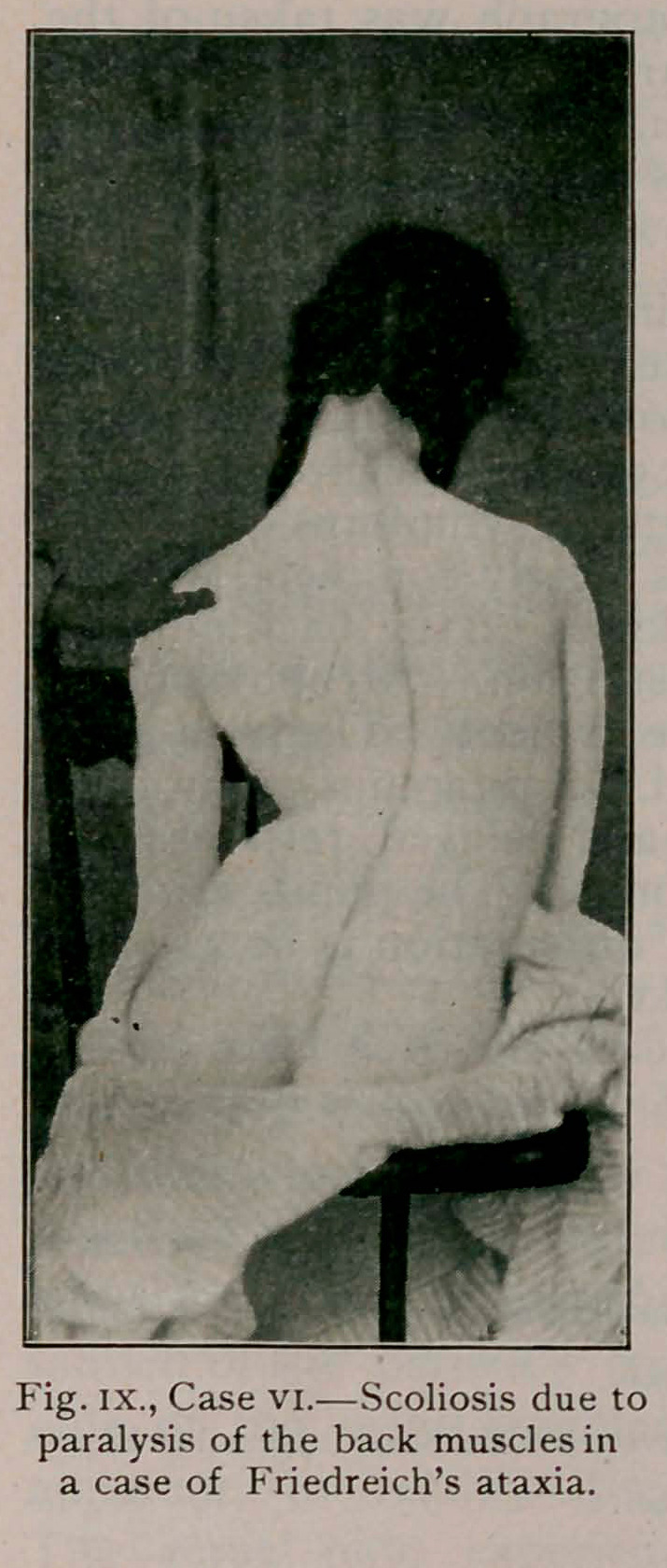# The Treatment of Rotary Lateral Curvature of the Spine1Read before the Buffalo Academy of Medicine, Section on Surgery, April 28, 1902.

**Published:** 1902-06

**Authors:** Prescott Le Breton

**Affiliations:** Buffalo, N. Y., Assistant orthopedic surgeon, Children’s Hospital, and assistant to the orthopedic surgeon, Erie County Hospital.


### The Treatment of Rotary Lateral Curvature of the Spine.[1]

By PRESCOTT LE BRETON, M. D., Buffalo, N. Y.,

Assistant orthopedic surgeon, Children's Hospital, and assistant to the orthopedic surgeon, Erie County Hospital.

THE marked improvement in the methods of diagnosticating and treating disease during the latter part of the last century has largely been due to a better understanding of etiology and pathology, an illustration of cause and effect. Laboratory research has sharpened clinical observation and as medicine becomes daily more scientific, our patients receive the benefits of the practical results. Although lateral curvature of the spine is not a disease, it is a matter of serious import to one suffering from this deformity. The great frequency of curvature, it being present in over 1 per cent. of growing children, according to Drachmann, and the probability of increase of distortion, warrant the investigation of its etiology and pathology with the aim of improving the methods of treatment.

During the past twenty-five years this branch of orthopedic surgery has received considerable attention and this paper has

1. Read before the Buffalo Academy of Medicine, Section on Surgery, April 28, 1902.

for its object the outlining of the treatment as it exists today in contradistinction to the obsolete methods formerly in vogue. That the prognosis has changed may be seen from the figures of Schulthess,[1] who has recently recorded a high percentage of favorable results—92.9 per cent of his cases out of a total number of 618 patients.

Before entering on the discussion of treatment a few words as to the method of examination for determining the kind of

Fig. I., Case I.—Lateral deviation of the spinous processes and rotation of the vertebræ. See fig. II. and III.

Fig. II., Case I.—Same patient as in fig. I. after nine months' of treatment by exercises and correction in the machine.

curve present, and as to the best means of recording progress in the case are indispensable, as these matters bear directly on the subject. The patient must be stripped to the waist and must stand with the clothes lowered until the entire lumbar region and the crests of the ilia are bare. Inspection of the back in good light detects readily a projection of one hip or of one shoulder, a lack of symmetry in the position of the scapulæ or a general inclination of the trunk to one side, one arm perhaps resting against the hip while the other swings clear of the body, as in fig. I.

The finger tracing over the tips of the spinous processes from cervical region to sacrum will find lateral deviation from the middle line of the body, if present. As the patient bends forward from the hips, holding the spine steady, the profile of the ribs comes into view, as in fig. VIII., and this position is the best for detecting rotation of the vertebræ which inevitably causes a prominence of the ribs on one side and a sinking on the other. Rotation, even to a slight extent in this horizontal position means a a fixed curve, *i. e.*, one in which structural changes exist.

Fig. III., Case I.—Same patient showing correction obtained in the pressure machine.

Testing the mobility of the different sections of the spine is next in order. By placing successively one or two thin books under one foot as the patient stands with extended knees, we tilt one side of the pelvis upward and produce a compensatory curve in the lumbar region. If lumbar curvature is present with convexity to the left, raising the pelvis by a book under the left foot will diminish the curve, but a book under the right foot will increase it. If a lumbar curve can be corrected or overcorrected by this means it is a flexible curve.

To test the motions of the dorsal spine the patient must clasp the hands high above the head and bend forward, backward, and to the right and left sides while the pelvis is steadied by the examiner. The patient should try to make the bend as high up as possible, not in the lower lumbar region. Inspection of the back during these movements gives information as to the character of the curvature. If stiffness and slight changes only in contour follow these efforts, it is positive that changes in the bones, ligaments and muscles have occurred, a condition calling for special treatment. A normal child can clasp the hands high above the head without increase of the normal lumbar lordosis; a round-shouldered child cannot. Another means of testing mobility is to draw the patient up in a Sayre's suspension apparatus, or in lieu of this to have an assistant place the palms of his hands under chin and occiput and draw the patient up from a sitting posture. The spine will then straighten as much as its conformation will permit. Hanging by the hands is an inferior test, because of the attachments of the scapular muscles to the spinous processes of the vertebræ. A final test of mobility is to place the patient in the pressure machine (see fig. III.), to discover to what degree direct force will overcome the deformity. Experiments have proved that the spine is governed by the same laws as flat, flexible rods, and that neither lateral deviation nor rotation can exist to any degree alone. Each is part of one compound movement.

Henry J. Bigelow summed up the matter thus in the Boylston prize essay for 1844:

> The principle of torsion is illustrated by bending a flat blade of grass or a flat, flexible stick in the direction of its width. The center immediately rotates upon its longitudinal axis to bend flatwise in the direction of its thickness. In the same way the spine, laterally flexed, turns upon its vertical axis to yield in its shortest or antero-posterior diameter.

A graphic method of recording cases is essential. Hence photographs, although open to objections, are largely used. Two photographs of each case at the beginning of the treatment should be taken, one in the upright position to indicate lateral deviation of the spinous processes, and one of the patient bending forward to indicate rotation of the vertebræ with distortion of the ribs. Another graphic method is to mold transversely across the back of the recumbent patient a thin strip of sheet lead, the center of the strip overlying a certain spinous process. The lead is removed, placed upon paper and the outline drawn by a pencil, the lead acting as a ruler. Another tracing is secured at another point and so on. Months later this can be repeated and changes in the contour of the back are shown by the variations in the curves on the paper.

Weigel has invented an apparatus which reproduces exactly the outline of the back. It should be a matter of routine to measure the length of the legs of each case and if unequal, to alter the thickness of the soles of the shoes. A record should be kept of the height and weight, the expansion of the chest, and the circumference of the forearm, arm and leg, because a marked increase in all these measurements follows the gymnastic work. A spring balance fastened to the wall may be made to measure the strength of individual sets of muscles. A general examination of the heart, lungs and other organs must precede exercises and forcible correction in order that latent disease may not be aggravated. The writer has seen cases of endocarditis and suspected tuberculosis in which active measures were contra-indicated.

*Preventive Treatment.*—Just as modern methods of treating disease are dealing more and more with prophylaxis, so in lateral curvature preventive treatment is becoming of great importance and is directed chiefly at regulating the posture of school children and providing for them suitable seats and desks, such as are described by Staffel.[2] Improper attitudes, standing frequently on one foot, writing in a twisted position, and sitting with the spine flexed will eventually produce distortion in pupils in whom there exists that predisposing cause of curvature, *i. e.*, inherent weakness and predisposition to deformity, or in other words, "lessened resistance to unfavorable conditions." As curvature almost always begins while the spine is in flexion, calisthenics, including exercises for extending the spine are of great service. Physicians should be careful to examine the back of any child concerning whom the mother speaks of a projecting hip or shoulder, or says, "she is getting one-sided." Corsets and articles of clothing that constrict the trunk are to be avoided.

It has been proven that it is weight obliquely applied to the spine that causes rotation and lateral deviation.[3] Weight equally applied causes antero-posterior bending only. Inquiring as to what may cause the superincumbent weight of the head and shoulders to fall obliquely we come to the question of asymmetry of the body. Everyone knows of the asymmetry found in nature in all living beings and in corresponding parts of the same body. In the human race asymmetrical position of the head from ocular defects has caused curvature; also asymmetry of the pelvis, first described by Barwell, and unequal thickness of the vertebræ found by Schulthess.

Fig. IV.—The screw pressure machine, described under treatment, section 4.

It is, however, to asymmetry of the legs that the writer would call special attention, a feature not emphasised as it deserves to be. Bradford and Lovett very truly remark that deformity from this cause is not invariable, but when present it should be corrected. The following table from their surgery gives an idea as to the frequency:

| Number of cases examined. |
|---|
| 21 |
| 230 |
| 30 |
| Total . . 281 |

| Number of cases showing unequal length of the lower extremities. |
|---|
| 17 |
| 62 |
| 28 |
| Total . . 107 |

In January, 1902, a lady, aged 30, was referred to the writer. She suffered from an S-shaped scoliosis of long duration, which had been treated by leather jackets and electricity for several years without improvement. One surgeon had informed her that a slow-growing tumor was present in the left lumbar region.

There was evident on examination a marked rotation of the lumbar vertebræ without much lateral deviation (a condition recalling the famous case of the physician reported by Adams in 1882, in whose back a tumor which was pronounced a cold abscess by the ablest surgeon in England, proved at autopsy to be the transverse processes of the lumbar vertebræ, rotated backward on the left side.) On raising the patient in the Sayre's suspension the spine straightened and the tumor vanished instantly, returning as the feet again supported the weight of the body. As she sat down it was noted that the curvature almost disappeared, hence she was asked to stand with the left foot on a book, and it was soon evident that a book three quarters of an inch in diameter completely corrected the deformity. Measuring from the anterior spine to the inner malleoli, the length of the right leg was found to be $32\frac{1}{2}$ inches, the left, $31\frac{3}{4}$. Questioning elicited the fact that in her 17th year she had had an attack of "brain fever" and ever since then "the left foot had not come down so solidly as the right." She was told to have the sole of the left shoe increased and at her next visit correction was evident, and she stated that her clothes fitted perfectly over her hips. Patients particular about their appearance may have the inner sole alone thickened.

John Hilton[4] in his famous "Rest and Pain," details his experiences in these words:

> I may add that I have seen so many of these cases, that I am persuaded that they occur not infrequently, and that they are usually overlooked. Thus I have seen many patients wearing spinal supports, in order to correct a lateral curvature, when the deformity might have, and has been subsequently, corrected by placing within the shoe or boot a piece of cork thick enough to compensate for the shortness of the less developed limb.

This is undoubtedly an exaggerated view but the fact remains that it is important to recognise and correct this asymmetry in the small number of cases in which it is present.

*Treatment.*—The following means present themselves for consideration, one combination being adapted to one case, another for another case: (1) Recumbency and suspension in the Sayre's apparatus; (2) special gymnastics; (3) support by corrective jackets; (4) forcible correction by the pressure machine; (5) electricity, massage and general treatment.

1. Recumbency was the chief and almost the sole agent in the hands of the older surgeons, and Adams cites many cases in which it did great harm, the patients having remained in bed indefinitely waiting for nature to correct the deformity. Recumbency is valuable, however, as an adjunct. Until a patient has become so strengthened by exercises that she can voluntarily hold her corrected position all day, she should have ten hours' rest at night and an interval of rest on the sofa after each midday meal. For a very young child with a rachitic curve, recumbency, manual correction, massage and feeding form the essentials of treatment.

Fig. v., Case II.—A mild grade of deformity producing unequal shoulders and hips.

Suspension in the Sayre's apparatus once or twice daily may be employed, the weight of the body tending to correct the deformity. In the case of rigid curves the force is ineffective and direct pressure in the machine is necessary to restore free passive motion.

2. In a previous article[5] the writer has dealt with the question of medical gymnastics and has mentioned the fact that the exercises must be ordered to suit each individual case as no two cases are exactly alike. Gymnastics alone suffice to cure the great majority of curvatures that are flexible, and they enable patients to retain whatever improvement in flexibility results from forcible correction in the pressure machine. Patience and perseverance are essential and although many require but three to six months of work a much longer time may be indicated. It is important to give not only special exercises directed at the spine, but a certain amount of general calisthenics to improve muscular tone (Teschner.) Ordinarily, flexion of a distorted spine increases the deformity, hence the value of such motions as will hyperextend the spine, as Lovett has pointed out.[6] Exceptional cases, however, which follow Whitman's second type[7], where the curve has originated in the lumbar region flattening the normal lumbar lordosis and producing a compensatory curve in the dorsal region with flattening of the normal dorsal kyphosis, should avoid hyperextension. Case 4 (see fig. 7) illustrates this very well and her exercises were beneficial when the spine was maintained slightly flexed.

3. When the plaster-of-Paris jacket was introduced for the treatment of Pott's disease it also was heralded as an efficient method of overcoming lateral curvature, but disappointment ensued.

In Pott's disease it is serviceable because it prevents increase of deformity and affords rest to the spine while nature cures the diseased process by ankylosis. In lateral curvature the spine is held in its distorted position by a jacket, the muscles and ligaments become structurally shortened and the muscles waste for want of exercise. The poor results obtained have led to a change in treatment and these jackets are now of service in selected cases only. In paralytic patients firm support is of course essential (see case 6). The experiments of Wullstein, who produced lateral curvature in young dogs by holding them in plaster-of-Paris in deformed positions for months would seem to justify the treatment employed by some orthopedists who apply jackets to *young* children in the recumbent position, whose curvatures are held corrected by apparatus during the hardening of the plaster. A few months later a new jacket is applied and additional correction is obtained, the spine meanwhile growing in its new position.

The use of corrective jackets during a portion of each day depends on another principle, *i. e.*, to act as a temporary support until exercises have strengthened the muscles and to suggest constantly to the patient the necessity of voluntary effort to obtain the "key-note" position. They favor also adaptive changes in the bones while growth progresses. At the outset of treatment for a weak patient with a rigid curve, the writer usually makes a plaster-of-Paris form, then a solid cast of plaster, and having corrected the cast until the two sides are symmetrical, molds a leather jacket which conforms to the cast. This is worn by the patient a portion of each day only until she has learned to hold her keynote position, and has developed some power in the spinal muscles. That the jacket applies its corrective force exactly is shown on removal of the same by the reddening of the skin over the convexity and the paleness of the skin over the concavity of the ribs. The wearing of corrective jackets for selected cases as an adjunct to other treatment meets the approbation of orthopedists at the present time.

4. The use of force, skilfully applied, is not only a great aid to the surgeon at the beginning of treatment of certain conditions but oftentimes an absolute necessity. For example, one cannot hope to cure a rigid flat-foot, a falsely ankylosed joint, or a wrist stiffened by a previous Colles's fracture without using force until resistance to passive motion is entirely overcome.

Fig. VI., Case III.—A severer type with beginning rigidity and bony changes.

Free passive motion having been provided, mild therapeutic measures insure recovery. When a rigid and distorted spine is presented for consideration we have the same indications to meet—the resistance to passive motion must be overcome or improvement is impossible. The problem is difficult to solve owing to the complex nature of the parts and the motions involved. It must be admitted that the treatment of rigid lateral curvature is necessarily unsatisfactory, and that just so far as any case has developed changes in the bones, ligaments, cartilages and muscles, just so far will treatment be ineffective in correcting the deformity. In other words, improvement but not cure must be expected in proportion to the age of the patient and the degree of structural alterations. A girl 14 years old with a medium grade of distortion can be far more successfully cared for than a girl of 18, with a severe and old deformity. Many simple devices have been used for the forcible correction of curvatures and the restoration of free motion, but the objections to them are that they do not fulfil the requirements. The screw pressure machine, used by the writer, as seen in figure 3, meets the indications as scientifically as possible and it really accomplishes what the inventors claimed it should. The photograph describes it better than words. Its principle depends on the fact, proved by experiments on models and cadaver, that oblique pressure on the ribs causes rotation of the vertebræ and produces lateral deviation of the spinous processes when the cervical and sacral regions are held motionless. The machine has been extensively improved by Weigel, of Rochester.[8] It was introduced in its original form by Schede, of Hamburg, and Hoffa, and modified by Bradford and Brackett, of Boston. It consists of a large steel frame with adjustable attachments for the head, hands, pelvis and trunk. The rear horizontal semi-circular bar is swung backward, the patient steps to the center between the uprights and the bar is swung to and fastened. The head piece is adjusted and the patient raised on tiptoe, thus removing the weight of the head and producing traction upward. The hands aid in this by clasping the short bars above the head. The pelvic attachments immobilise the pelvis and may be swung on a vertical pivot to untwist a lumbar curve. The side bar is pushed inward to the left axilla and screwed tight, thus fixing the upper dorsal region. The various padded pressure plates are now brought into contact with the body, one of large size over the projecting ribs posteriorly opposite the apex of the curve where the greatest pressure is to be made, and another diagonally opposite, below the left breast. One is brought to bear under the right breast, one against the front of the right shoulder and one against the left shoulder posteriorly. Additional pads may be placed against the sternum and against the transverse processes on the convexity of a lumbar curve, if it is present.

The chest is now held firmly at every point except at the concavity of the dorsal curve where the ribs are depressed. As the screw is turned and the pressure increased on the convexity, the ribs yield on the opposite side and begin to bulge while the spinous processes approach a straight line. The screw is turned and the correction increased until the patient is rendered uncomfortable. The patient returns daily at first, later three times weekly for a twenty-minute correction, until flexibility has returned and the patient can in her exercises straighten the spine to the same extent as the machine.

This pressure machine is adapted for patients of all ages. It may be adjusted to a case in about three minutes and the patient may be removed in a few seconds. The pressure is uncomfortable, but never needs to be painful. Without this forcible correction the rigidity will not yield and no improvement can be looked for. Manual correction and simple pressure without correct counter-pressure in front allows the force to be dissipated and the whole stiff segment of the spine is thrust sideways as a unit. In the machine the pressure is followed up and the force is applied to the deformity itself. It is necessary for one to have practical experience for some time in the use of the machine to learn minor details and to become accustomed to the needs of different patients.

Under the heading of forcible correction a few words must not be omitted on a new use of the plaster-of-Paris jacket for fixed curves devised by Lovett.[9] He places the patient face downward on a modified Bradford frame with the thighs at right angles to the pelvis. This position flattens the lumbar curve which in turn will flatten the dorsal curve. The spine thus hyperextended and partially corrected is straightened further by bandaging the trunk to the sides of the frame, one bandage pulling on the convexity of the dorsal curve and the other on the convexity of the lumbar curve. The trunk is now encased in plaster. At the end of five days the jacket is removed and another applied in the same way exercising more force. Several jackets are thus rapidly worn and discarded, and then the patient is treated by exercises. Lovett claims that correction proceeds at a rapid pace by means of this preliminary use of jackets. He found that in the earlier jackets it was very easy to obtain an unbearable amount of corrective force, and that the

Fig. VII., Case IV.—Showing structural changes with considerable rigidity of the spine.

patients must become accustomed to a gradual increase of pressure.

5. Daily applications of faradic electricity to the muscles of the back may be given in addition to other means. When the deformity is due to anterior poliomyelitis, the weakened muscles are on the convex side and the electricity must be applied to them. Massage of the back after exercises is grateful to the patient and sea salt rubs in the morning are refreshing. The surgeon must be careful that the treatment is not overdone to the detriment of the general health of the patient and rest must be ordered after any temporary illness. The condition of the vital organs should be examined at intervals by the family physician.

Fig. VIII., Case V.—The profile of the ribs indicates very marked rotation and rigidity.

Curvatures due to empyema, sciatica and lesions of the spinal cord must be treated according to indications. When the lumbar region is chiefly at fault, partial correction can be obtained by thickening the sole of the shoe on the convex side and by the use of an inclined seat. Tenotomy of one or more muscles of the back as performed so energetically by Guérin, of France, and later by Sayre, has been entirely discontinued and finds no place in modern text-books. Hopelessly deformed patients may be relieved by mechanical support, massage and electricity. If great pain is caused by overlapping ribs in extreme cases resection of one rib is a simple expedient, as performed by Goldthwait and Painter, of Boston.[10] It may be repeated, in conclusion, that each patient deserves special study and for each there must be ordered that combination in treatment best suited to the individual case.

The writer has selected the following to illustrate different types of the same deformity:

Case I.—(Figs. I., II. and III.) aged 15. This patient entered the Children's Hospital in July, 1901, a flabby, weak, overgrown girl with a long dorsal curve and slight lumbar curve shown in fig. I. Rigidity was marked. Nine months later, while still under treatment, she was photographed again and the result appears in fig. II. To meet the objection that the improvement is only apparent, it may be said that on each occasion the girl was asked to hold herself straight as she would naturally while standing. One must see a case exercise to obtain an accurate idea of the result of treatment. She was a very unpromising patient at first and frequently became faint or vomited during her gymnastics. Her general health is now excellent and she has discarded her corrective jacket, but exercises daily and is stretched in the machine once a week. The same patient is seen in the machine in fig. III., and the line over the spinous processes was drawn after correction had been obtained.

Fig. IX., Case VI.—Scoliosis due to paralysis of the back muscles in a case of Friedreich's ataxia.

Case II.—(fig. V.) aged 16; referred by Dr. F. A. Drake, of Buffalo. A typical example of a mild flexible deformity with a prominent hip which called the attention of the mother to the back. The right leg was found to be three-eighths of an inch shorter than the left and a thickened sole aided considerably in correcting the curvature. As an example of what gymnastics will do in seven weeks, the following comparisons are given:

| | Measurements January 20. | Measurements March 14. |
|---|---|---|
| Chest at expiration | 25¼ inches. | 26¼ inches. |
| Chest at inspiration | 27¼ " | 29¼ " |
| Forearm | 7⅞ " | 8½ " |
| Arm | 8¼ " | 8⅝ " |
| Calf | 12⅛ " | 13 " |

Case III.—(fig. VI.) Aged 13, entered the hospital in September, 1901. One sister suffered from a severe type of lateral curvature. Bony changes and a beginning rigidity necessitated machine treatment in addition to exercise. Complete cure will be obtained in her case.

Case IV.—(fig. VII.) aged 16, referred by Dr. De Witt Hitchcock, of Oxford, Chenango County, N. Y. A very tall, thin girl with flattening of the normal physiological curves. A higher grade of deformity is evident and a complete correction is impossible. Considerable progress, however, has already been made in improving the flexibility of the spine.

Case V.—(fig. VIII.) A deaf mute, aged 15, referred by Dr. T. F. Dwyer, of Buffalo. The photograph was taken of the profile of the ribs to show the very marked rotation and rigidity continuing in the horizontal position. The worst deformities occur often in males, and this case is an example of what neglect of parents may cause.

Case VI.—(fig. IX.) Aged 18, an inmate of the Erie County Hospital. An illustration of a marked scoliosis, due to Friedreich's ataxia. One sister and one brother suffer from the complaint. At the age of 14, weakness of the lower extremities developed and gradually all of the typical symptoms appeared. Paralysis of the lower extremities is nearly complete, but the arms still retain some power. Weakness of the back muscles has caused the curve seen in the illustration. Atrophy of the muscles and marked incoördination are evident. The knee-jerks are absent and the sphincters unaffected. Nystagmus can usually be elicited. Sensation is inaccurate and delayed, response following an interval of about three seconds. The pupils are normal and instead of explosive speech articulation is hoarse and slow. Support is provided for by jackets.

BIBLIOGRAPHY.

1. Zeitschrift f. Orthopaed Chirurgie. Vol. 9, No. 3. Quoted by Phil. Med. Jour., March 15, 1902.
2. Orthopedic Surgery. Bradford and Lovett, p. 120.
3. Ibid. p. 97.
4. Hilton. "Rest and Pain," p. 240. Wood & Co. 1879.
5. Le Breton. Corrective Exercises for a Case of Lateral Curvature of the Spine. American Medicine, Feb. 1, 1902.
6. Lovett. Boston Med. and Surg. Jour., June 14, 1900.
7. Whitman. Orthopedic Surgery, p. 125.
8. Weigel. The Rational Treatment of Lateral Curvature of the Spine. Journal of Physical Therapeutics, Oct., 1901.
9. Lovett. Boston Med. and Surg. Jour., Oct. 31, 1901.
10. Painter. Phil. Med. Jour., Dec. 14, 1901.

122 North Pearl Street.

---

Keen (*American Journal of the Medical Sciences*, July, 1901,) in an article describing several operations for traumatic epilepsy in which he injured the lateral sinus, comments on the apparent harmlessness of the accident. No untoward results were noticed in any of the cases in which it occurred. He operated on one case in which a piece of bone embedded in the cerebral substance had escaped detection.

## Figures and Tables

**Fig. I., Case I. f1:**
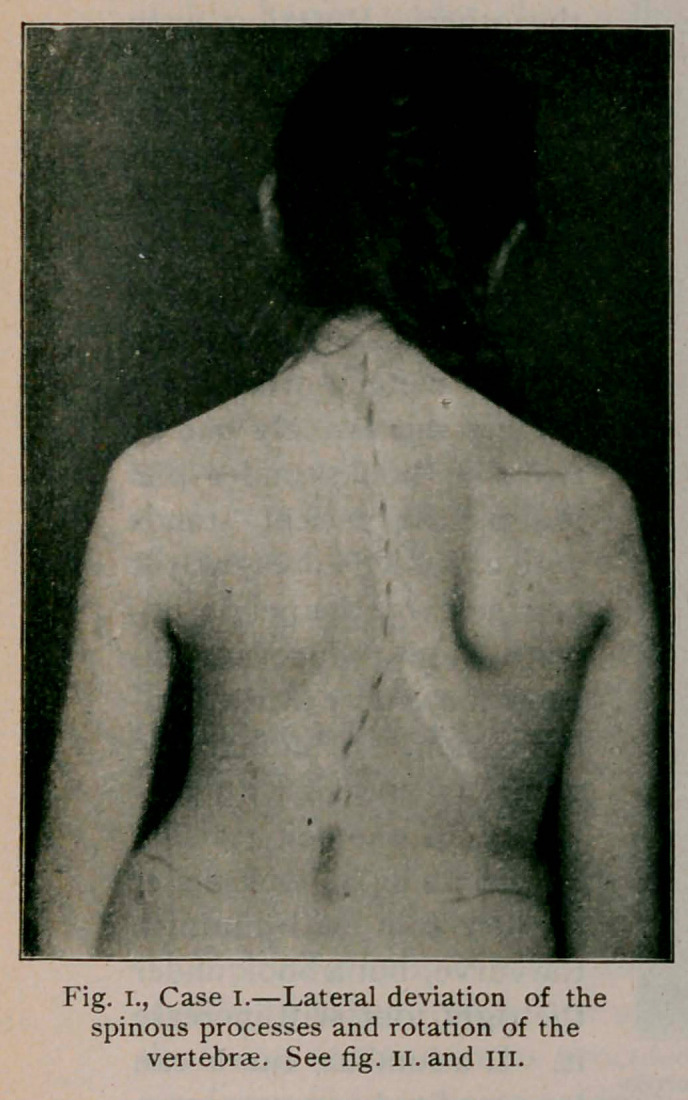


**Fig. II., Case I. f2:**
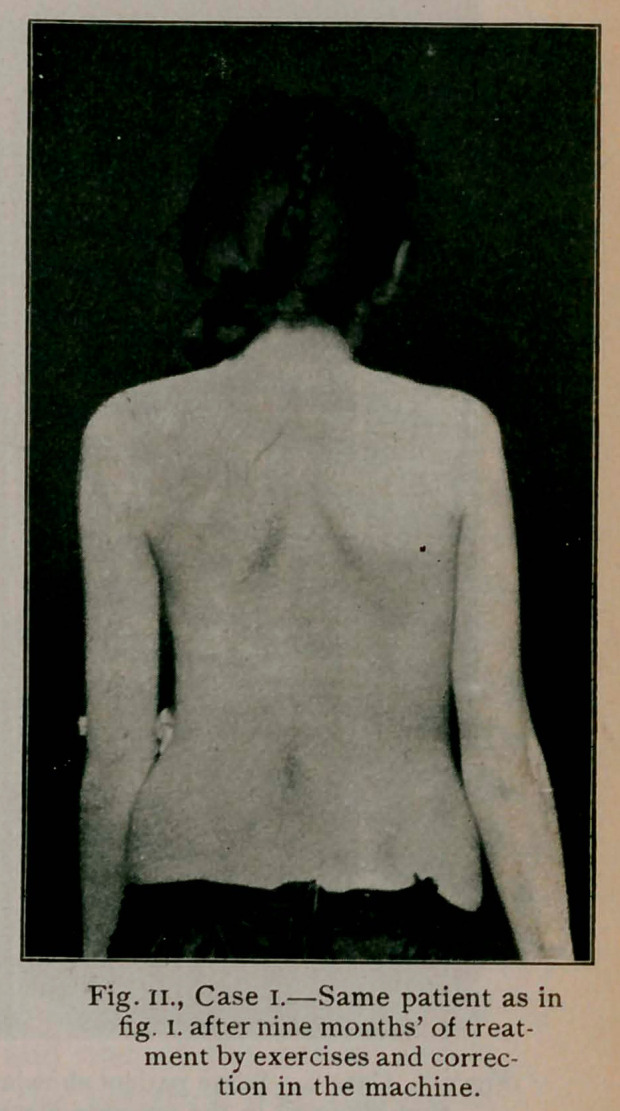


**Fig. III., Case I. f3:**
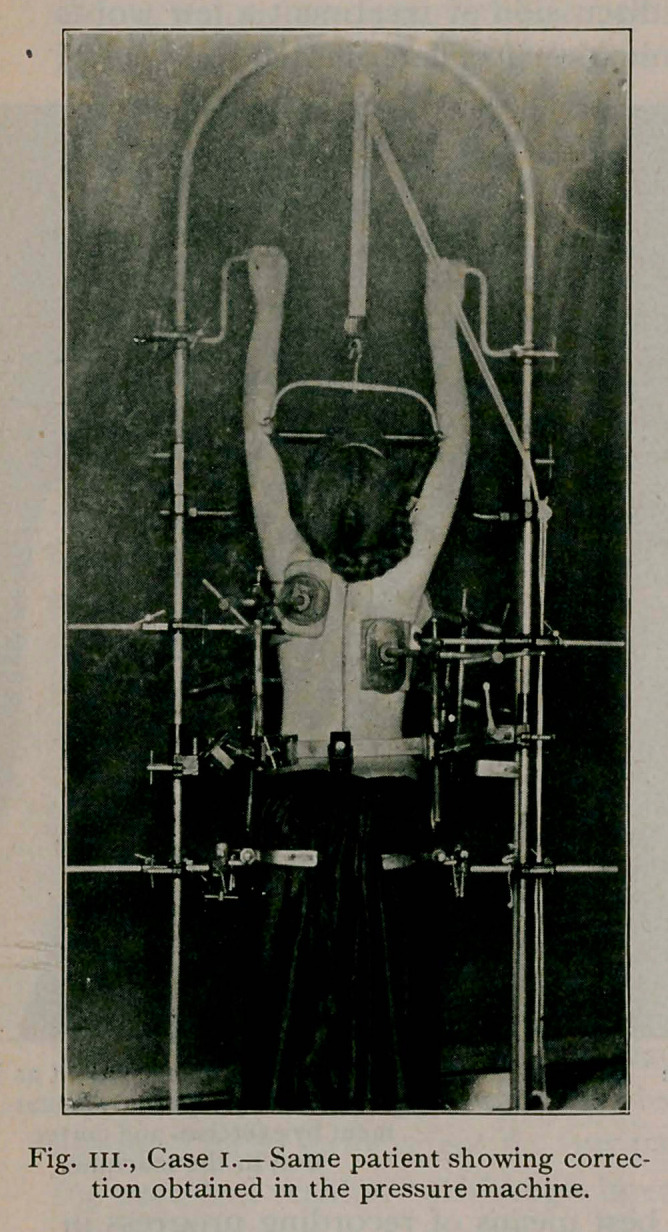


**Fig. IV. f4:**
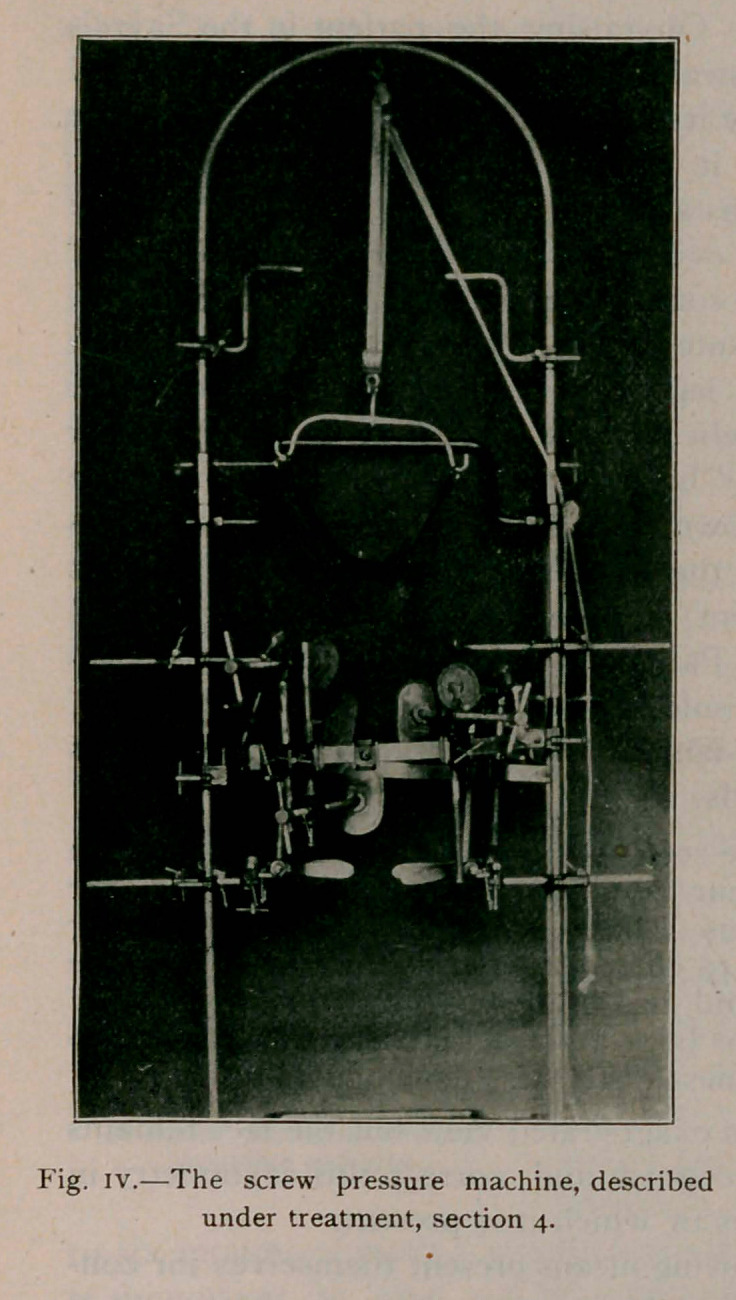


**Fig. V., Case II. f5:**
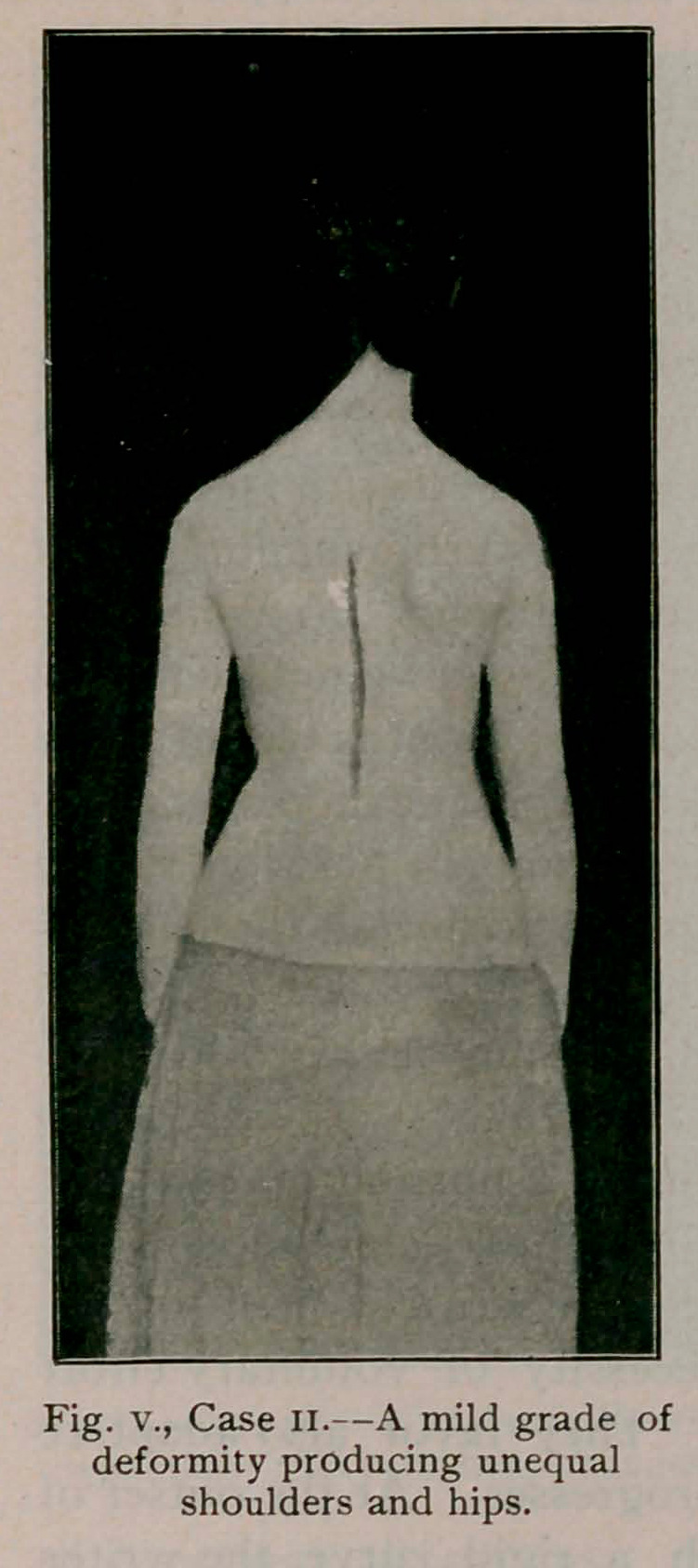


**Fig. VI., Case III. f6:**
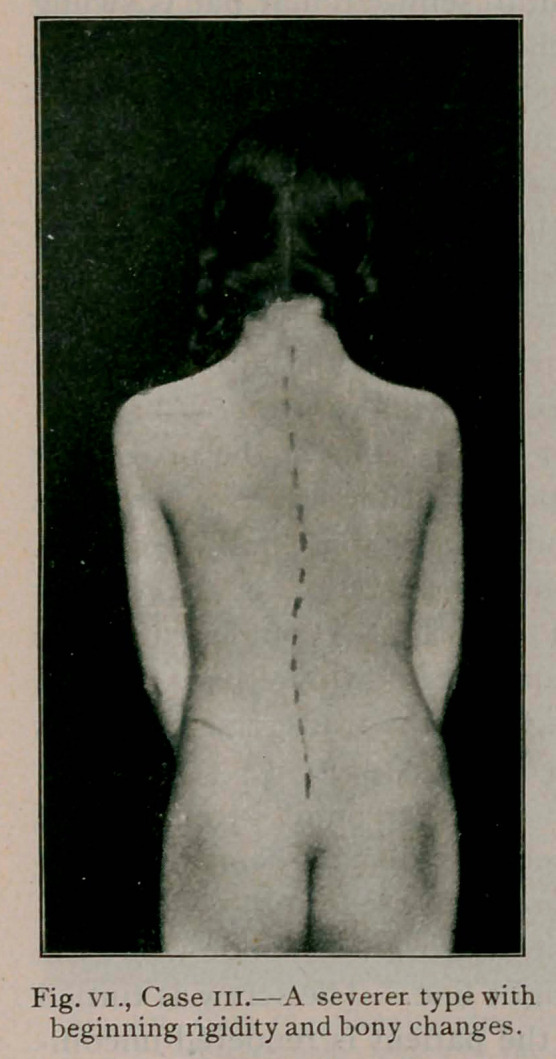


**Fig. VII., Case IV. f7:**
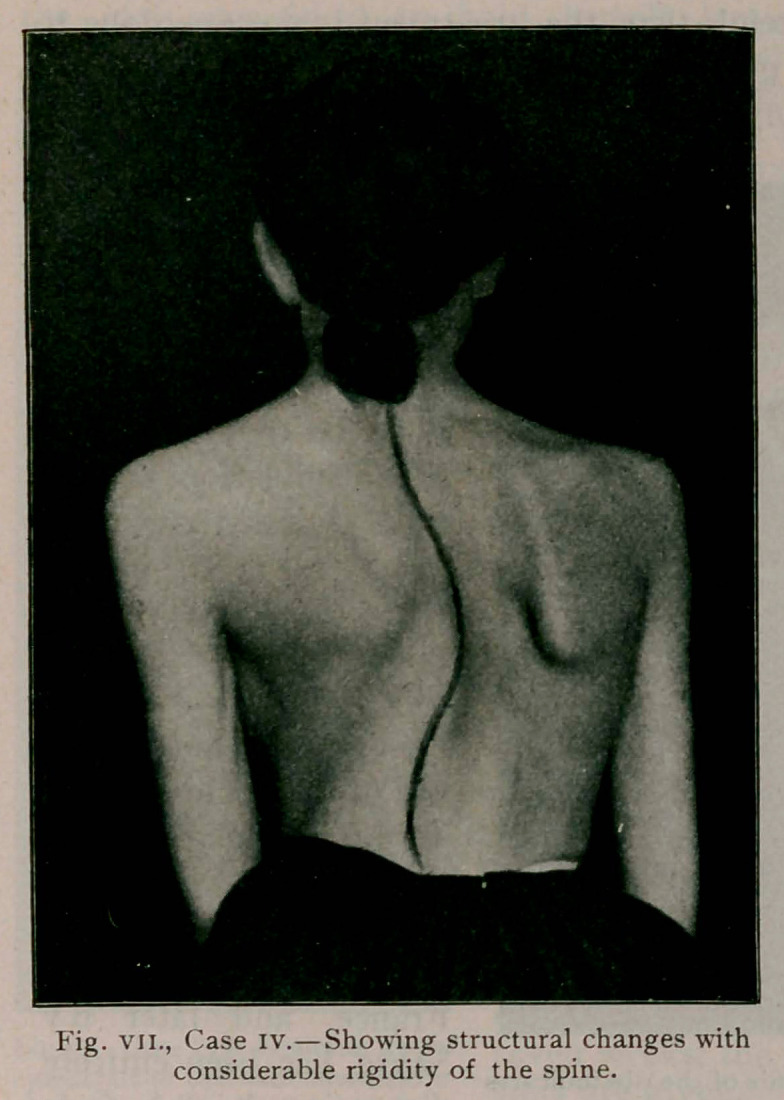


**Fig. VIII., Case V. f8:**
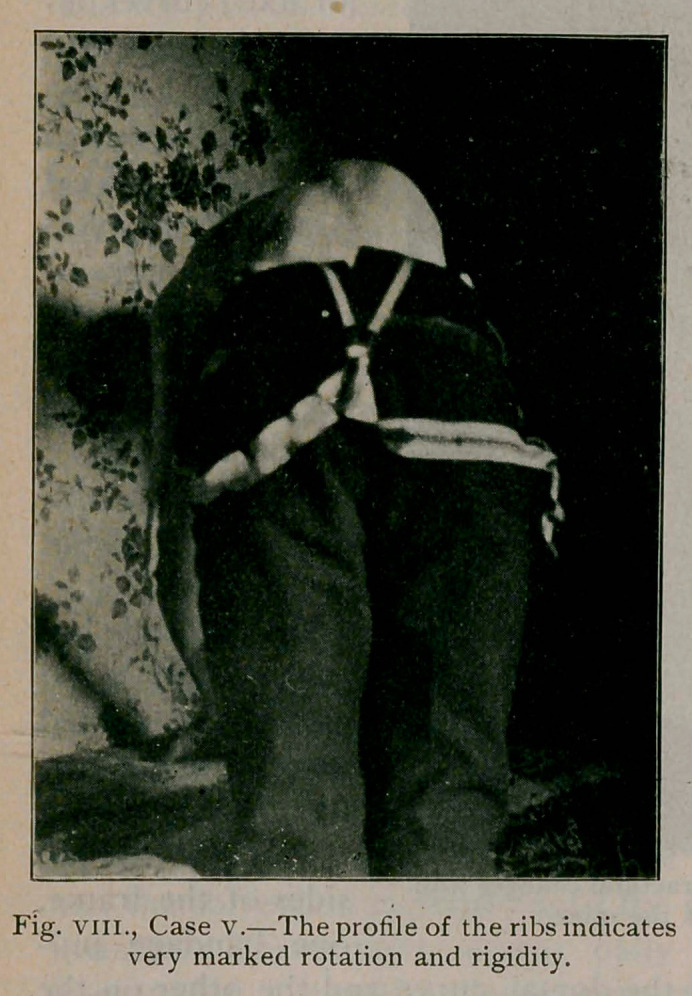


**Fig. IX., Case VI. f9:**